# Spontaneous intrathyroidal hematoma causing airway obstruction: A case report

**DOI:** 10.1097/MD.0000000000003209

**Published:** 2016-09-02

**Authors:** Corliss A.E. Best, Sandeep Dhaliwal, Samantha Tam, T. Hubert Low, Brian Hughes, Kevin Fung, S. Danielle MacNeil

**Affiliations:** Department of Otolaryngology, Division of Head and Neck Oncology & Reconstructive Surgery, University of Western Ontario, London Health Sciences Centre.

**Keywords:** airway obstruction, spontaneous thyroid bleed, thyroid hematoma, thyroid hemorrhage

## Abstract

**Introduction:**

Spontaneous thyroid hemorrhage is a rare occurrence that results in pain, discomfort, and occasionally compressive symptoms. Infrequently, extensive thyroid hemorrhage can result in a rapidly expanding hematoma resulting in airway compromise. This is a case of an otherwise healthy young woman, 3 months postpartum, with a slowly expanding spontaneous thyroid hemorrhage that measured at 7 × 5.5 × 5 cm by computed tomography. She ultimately required intubation to manage respiratory distress and subsequently a hemithyroidectomy for definitive treatment. The case presentation is followed by a literature review where known etiologies of thyroid hematoma including traumatic and nontraumatic causes, precipitating anticoagulation, and spontaneous rupture of branches of the external carotid artery are outlined. The potential links to pregnancy are explored. The roles of bedside thyroid ultrasound in the emergency department and lateral neck roentgenogram in diagnosis are explored. The importance of airway management and indications for conservative versus surgical treatments are discussed.

**Conclusions:**

This is a case of a spontaneous intrathyroidal hemorrhage, which progressed over days to ultimately cause airway compromise. It is imperative that physicians are educated on the appropriate detection and management of the potentially life-threatening spontaneous thyroid hematoma.

## 1 Introduction

Spontaneous thyroid hematoma is a rare cause of acute noninflammatory neck swelling that, in most cases, is self-limiting. Patients with acute hemorrhage present with a sudden onset of a cystic nodule, associated with local pain and discomfort, followed by rapid regression. Very rarely, hemorrhage into the thyroid gland can result in a rapidly expanding hematoma with resultant airway compromise. Massive intrathyroidal bleeding can potentially expand into the parapharyngeal and retropharyngeal spaces causing tracheal compression that can be life-threatening. Trauma is one cause of intrathyroidal bleeding in which the extent of the bleed and outcome is directly related to the severity of the inciting event.^[1]^ Nontraumatic causes of this entity include ruptured aneurysm or poststraining, as well as bleeding in coagulopathic states.^[2]^ Intrathyroidal bleeding may also occur spontaneously, and there are reports in the literature of patients with acute, massive hemorrhage into the thyroid gland with no confirmed etiology.^[3]^ We present a case of a spontaneous intrathyroidal hematoma, which progressed over days, resulting in airway compromise in an otherwise healthy woman who was 3 months postpartum. Management dilemmas of this clinical entity and its relationship to pregnancy will be discussed.

## 2 Case report

A 33-year-old woman presented with a spontaneous expanding intrathyroid hematoma causing acute airway obstruction. The hematoma occurred suddenly and caused gradual progression of symptoms that resulted in multiple emergency department (ED) visits with eventual airway compromise. The patient's account is presented in Appendix 1.

### 2.1 Clinical history

The patient first presented to the ED with a chief complaint of anterior neck discomfort, neck stiffness, and neck swelling that had developed over the course of the day. She had no history of preceding trauma, coagulopathy, or use of anticoagulants. She was sent home on oral antibiotics. Three days later, she presented to the ED for a second time, noting ongoing neck pain, increased stiffness, and increased swelling. Review of systems was negative. She was discharged home with an outpatient referral for a thyroid ultrasound. The thyroid ultrasound, which was completed 4 days after her initial presentation, demonstrated an asymmetrically enlarged left thyroid lobe containing a heterogenous hypoechoic mass measuring 7.7 × 5.1  × 5.6 cm, suspicious for a hemorrhage into a preexisting thyroid nodule. An outpatient referral to Otolaryngology was made. She returned to the ED for a third time to report worsening of neck swelling and pain and was discharged home to await Otolaryngology consultation. Two days later, the patient presented to the ED for a fourth time with a significant increase in neck swelling, pain, dysphagia, and for the first time, respiratory distress. Past medical history was significant for increased body mass index (BMI). She was 3 months postpartum for a planned cesarean section at 38 weeks with her second child. Her only medication was an oral contraceptive pill. There was no personal or family history of thyroid disease.

### 2.2 Physical examination

**Figure 1:**
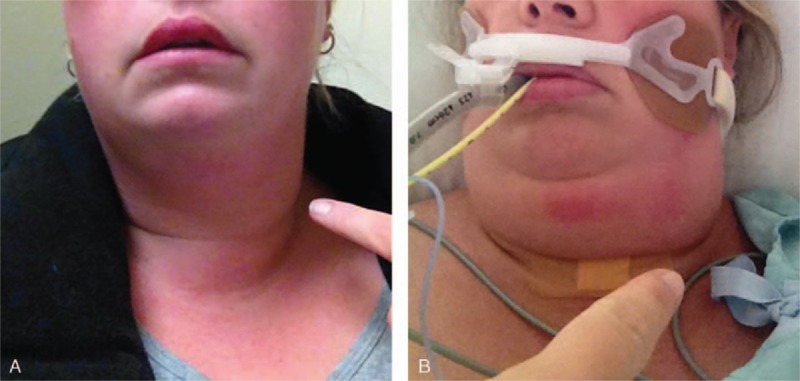
(A) Patient photograph at initial presentation to emergency department. (B) Patient photograph immediately following intubation after progression to airway compromise.

On initial ED presentation, the patient was noted to have edema, erythema, and tenderness on palpation of the left side of her neck, which progressed over subsequent ED visits (Fig. 1A). On her fourth ED presentation, the patient was noted to be stridulous while supine. At this point, she was intubated for airway protection in the operating room by the anesthesia service. There was no significant narrowing of the upper airway. On admission to the intensive care unit (ICU), examination of the neck revealed palpable fullness near the left thyroid and significant submandibular swelling bilaterally (Fig. 1B). She was febrile on presentation to the ICU, but otherwise her vital signs were stable.

### 2.3 Diagnostic investigations

**Figure 2:**
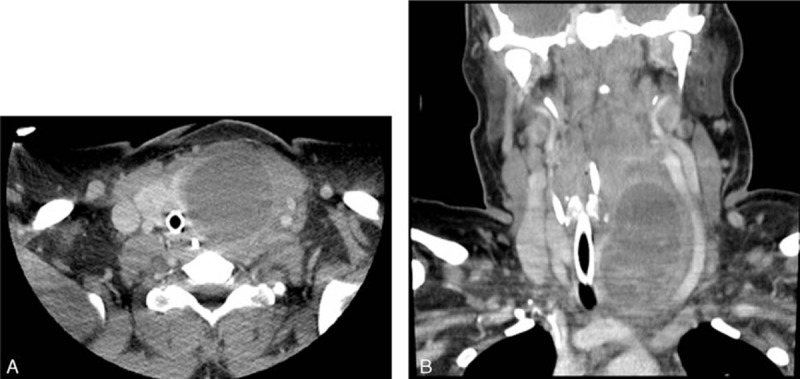
(A) Contrast-enhanced axial CT scan demonstrating left intrathyroidal hematoma. (B) Contrast-enhanced coronal CT scan demonstrating tracheal deviation and compression from left intrathyroidal hematoma.

Following intubation, a computed tomography (CT) scan was performed (Fig. 2A and B) revealing a 7 × 5.5  × 5 cm large ovoid complex lesion in the left thyroid lobe with deviation of the larynx. The findings were highly suspicious for hemorrhage into the left lobe. Blood work demonstrated a downward trend in hemoglobin (108–97 g/L). Blood cultures were negative for infectious organisms.

### 2.4 Management

After securing the airway, the patient was transferred to a tertiary care center. After obtaining informed consent, a left hemithyroidectomy was completed. Intraoperatively, a hematomatous nodule was found on the anterior aspect of the thyroid gland. Extensive hemorrhage was noted within the strap muscles and left thyroid bed extending to the carotid sheath. There was no acute bleeding while in the operating room. A hemithyroidectomy was performed in the standard fashion with careful dissection of the recurrent laryngeal nerve. Antibiotics were continued and the patient was started on dexamethasone to reduce airway edema.

The patient was intubated for 72 hours postoperatively until there was a cuff leak. Postextubation endoscopy revealed a patent airway, and normal vocal cord function.

The pathology report described the gross specimen as a tan-brown, smooth lobe containing a solid, red-brown, granular mass measuring 7.0 × 4.0  × 4.0 cm. The majority (90%) of the thyroid lobe was composed of necrotic thyroid follicles and hemorrhagic necrotic material, with no evidence of malignancy.

Follow-up ultrasound at 3 months revealed no nodules on the contralateral side and a normal left postthyroidectomy bed.

## 3 Discussion

In this paper, we describe a spontaneous, intrathyroidal hematoma that progressed over days eventually causing airway obstruction. There are several reports in the literature of rapidly expanding thyroid hematomas causing airway compromise with clear precipitating factors, such as anticoagulation, trauma, or a history of thyroid nodules.^[4]^ Several issues may have led to a delay in diagnosis for this patient, including an elevated BMI with increased neck girth and no clear inciting factor. This case also highlights the importance of prompt imaging in patients with atypical presentation of a neck mass.

Trauma as a cause for spontaneous hemorrhage in the thyroid gland has been well described. Etiologies can include blunt trauma or increased vascular pressure due to excessive Valsalva maneuver.^[1]^ In the majority of the reported cases, a thyroid pathology was either known prior to the hemorrhage or discovered on final histopathology examination.^[1,2]^ The patient presented here had no reported inciting trauma and also no known or discovered thyroid pathology. Histopathological diagnosis was difficult to ascertain due to extensive necrosis; however, the contralateral lobe on ultrasound was unremarkable.

Chronic anticoagulation as a cause for thyroid hemorrhage has also been reported. Spontaneous bleed into either a goiter or thyroid nodule has been reported in patients who are anticoagulated with warfarin, aspirin and for hemodialysis.^[5]^ This patient had no previous history of coagulopathy nor did she report prescription or nonprescription use of anticoagulants.

Rare neck hematomas secondary to spontaneous rupture of branches of the external carotid artery have also been documented.^[6]^ The CT scan in this scenario will reveal a hematoma in the region of the external carotid artery or adjacent to the thyroid lobe. In comparison, the CT scan of our patient's hematoma revealed a well-circumscribed, hypoechoic mass with a rim of thyroid surrounding the hematoma. There are distinct differences in imaging findings when comparing an intrathryoidal bleed versus an extrathryoidal bleed.

There are several unique factors in this case. First, the increased BMI of this patient may have made initial identification of hematoma more difficult by masking presentation on visual inspection and limiting the extent of palpation. Second, our patient was 3 months postpartum. The thyroid can increase 30% in size during pregnancy.^[7]^ Pregnancy has also been associated with new thyroid nodule formation.^[7]^ There have been 2 case reports involving airway obstruction caused by goiters in pregnant women.^[8,9]^ Three articles were identified reporting rare spontaneous rectus sheath hematoma.^[10]^ We were unable to identify any reports involving spontaneous thyroid hematoma formation in pregnancy or in the postpartum period. In this case, it is unknown whether her recent pregnancy had any effect on thyroid nodule formation and subsequent thyroid hematoma.

Ultrasound, lateral neck roentgenogram, and CT scans can be used in combination with clinical presentation to aid in the diagnosis of a neck mass. There may be a role for bedside ultrasound in the emergency department with acute neck swelling to rapidly identify hematoma.^[11]^ In this case, ultrasound was scheduled in an outpatient setting, which may have prolonged diagnosis and follow-up care. Although not used in this case, anterior–posterior and lateral neck roentgenogram can be used to assess for tracheal deviation and widening of the prevertebral space.^[12]^ Investigations for bleeding or clotting disorders, thyroid-stimulating hormone (TSH), antibodies, and corrected blood calcium levels should be completed when ruling out endocrine thyroid or parathyroid etiologies.^[5]^

In cases of acute neck swelling, airway management is critical. Conservative management with close monitoring, antibiotics, and steroids are appropriate for patients with no airway symptoms. Repeated sonographic imaging may be appropriate in patients who are treated conservatively.^[13]^ Antibiotics should be considered in the presence of pyrexia, especially in immunosuppressed patients. It has been suggested that upper airway hematomas secondary to anticoagulation are caused by localized infection with subsequent vasodilation, however, this theory is unproven.^[14]^ There is also little evidence to suggest that systemic steroids are beneficial in the setting of upper airway hematomas, although they have been used in other case reports.^[15]^ Fine needle aspiration can be attempted as first-line therapy in stable patients. However, this may be difficult if the bleed is slow and gradual, as the blood can clot, as was the case with this patient. Intubation is required if symptoms of airway obstruction are present, which may be difficult secondary to venous congestion and edema of the airway. Airway management may necessitate a trained anesthesiologist and surgical backup.

In select cases, surgical intervention is required. Persistent respiratory distress and dysphagia that is not relieved by needle aspiration are both indications for surgical interventions.^[4]^ Evacuation of the hematoma alone has been reported in extrathyroidal hemorrhage.^[6]^ In this case, a hemithyroidectomy was required due to the intrathyroidal nature and large size of the hematoma measuring 7 × 5.5  × 5 cm. Another case report of sudden massive neck swelling due to hemorrhage of a thyroid adenoma measuring 6 × 7 × 12 cm was also managed successfully with a hemithyroidectomy.^[9]^

## 4 Conclusion

We present a rare case of an otherwise healthy woman with a spontaneous intrathyroidal hemorrhage, with no preceding thyroid disease or history of trauma, leading to airway compromise. A hemithyroidectomy was carried out as definitive treatment. A review of the literature shows that thyroid hematomas can occur traumatically, nontraumatically, or spontaneously. Investigations including the role of bedside ultrasound in the emergency department and prompt x-ray were discussed. Treatment options including airway management and hematoma treatment, both conservative and surgical, were outlined. The authors recommend imaging modalities including bedside point of care ultrasound and CT for any presentation of atypical neck swelling. If airway compromise develops, the airway must always be secured by the anesthesia service. In conclusion, it is important for any medical practitioner to be aware of an unusual presentation of a spontaneous intrathyroidal hematoma with the potential for eventual airway compromise.
